# Towards Functional Fertilisers: Feed Composition Shapes Microbial Community Structure and Viability in Black Soldier Fly ( *Hermetia illucens* ) Frass

**DOI:** 10.1111/1462-2920.70249

**Published:** 2026-02-05

**Authors:** Daniel Kreft, Sabine Hurka, Friscasari F. Gurusinga, Till Röthig, Andreas Vilcinskas, Dorothee Tegtmeier

**Affiliations:** ^1^ Branch for Bioresources Fraunhofer Institute for Molecular Biology and Applied Ecology (IME) Giessen Germany; ^2^ BMFTR Junior Research Group in Bioeconomy (BioKreativ) “SymBioÖkonomie” Giessen Germany; ^3^ Institute for Insect Biotechnology, Justus Liebig University Giessen Germany

**Keywords:** biodiversity, circular economy, insect manure, metabarcoding, regulation (EU) 2021/1925, sustainability

## Abstract

Black soldier fly larvae (BSFL) are increasingly valued as a sustainable protein source for aquaculture and can be reared on local industrial side streams, enhancing their environmental and economic benefits. The resulting frass—a byproduct of larval excreta and residual feed—shows promise as an organic fertiliser. In addition to its nutrient content, frass contains microbial communities that may enhance plant growth through phytohormone production, nitrogen fixation, and organic matter turnover. Yet, the roles of feed composition and thermal hygienisation in shaping these communities remain underexplored. This study examined the impact of five feed substrates, including industrial side streams and a control diet, on frass microbial composition, and assessed responses to thermal treatment. Feed nutrients were characterised, and microbial communities profiled using amplicon sequencing. Viable populations were quantified via culture‐based methods, with bacterial isolates taxonomically classified. Feed type was the dominant factor influencing frass microbiota, with distinct communities reflecting substrate nutritional profiles. High‐fibre diets promoted fungal diversity and abundance, while high‐protein feeds enriched specific bacterial taxa. Thermal hygienisation had a heterogeneous effect on viable counts but minimal impact on overall community structure. These findings support microbiome‐informed feed design to tailor frass microbial profiles for enhanced biofertiliser function in sustainable agriculture.

## Graphical abstract

Frass derived from black soldier fly larvae reared on various industrial side streams was investigated in both untreated and hygienised (1 h at 70°C) condition. Hygienisation efficacy was evaluated by determination of viable microbial counts, using both selective and non‐selective cultivation media. Microbial communities were analysed using culture‐independent amplicon sequencing and culture‐dependent Sanger sequencing of bacterial isolates.

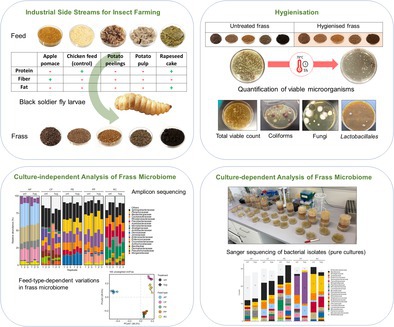

## 1 Introduction

The growing global population and changes in dietary preferences are challenging current and future food production. This is amplified by global change, including the loss of arable land and conflicts between agriculture and biodiversity protection. Insects have gained interest as alternative and sustainable animal protein (Abd El‐Wahab et al. 2021; Hermansen et al. 2024; Klüber et al. 2024; Calvo‐Baltanás et al. 2025). They can provide high‐value animal protein at fast production rates, valorise underused substrates, be farmed with little requirements to land and water, and provide additional side products including lipids, valuable molecules (e.g., chitin/chitosan and antimicrobial peptides), and frass. Insect frass is defined as a mixture of insect excrements, insect body parts, feed leftovers, and dead insects, which has gained considerable attention as a locally producible organic fertiliser. This holds particularly true as prices for mineral‐based fertilisers have recently increased substantially (EUCOM 142, 2011; EUCOM 1925, 2021; Hebebrand and Laborde Debucquet 2023; Caparros Megido et al. 2024; Cattaneo et al. 2024).

The effect of fertilisers, including those derived from insect frass, primarily depends on their macronutrient content and composition. These include nitrogen (N), phosphorus (P), and potassium (K), ratios of which can be optimised according to the target plant species. Additionally, micronutrients, including iron (Fe), manganese (Mn), copper (Cu), and zinc (Zn), are central for plant growth (Welch and Shuman 1995; Gärttling and Schulz 2022). Further, certain plant growth‐promoting microbes may have positive effects. Microbial activities that support plant growth include nitrogen fixation, nutrient solubilisation, and the production of phytohormones, for example (Pii et al. 2015; Del Orozco‐Mosqueda et al. 2023). Insect frass contains associated bacterial and fungal microbiomes, which presumably contribute to the plant promoting pathways (Barragán‐Fonseca et al. 2022). The frass is considered a microbial pathway connecting insect gut and plant roots (Poveda 2021). Particularly, insect gut‐associated microbiomes are well studied. They assist in insect growth and development by supporting digestion, health, and reproduction (Engel and Moran 2013; De Smet et al. 2018; Klüber et al. 2024). The gut microbiota of Black soldier fly larvae (*Hermetia illucens*, BSFL), for example, is driven by the feed composition and is believed to play an essential role in the utilisation of complex feed components like fibre (Gurusinga et al. 2026). By comparison, there is a paucity of data on the frass‐associated microbiomes, particularly regarding how different feeds affect the frass' composition. Further, to obtain approval for fertiliser in the EU, insect frass needs to undergo thermal hygienisation to reduce the number of harmful microorganisms such as coliforms (EUCOM 142, 2011; EUCOM 1925, 2021).

There is limited information on how heat treatment alters microbial taxa in frass that are commonly associated with soil functioning, including putative plant growth–promoting bacteria, lactic acid bacteria, and fungi, and whether these taxa remain viable after processing. Previous studies report variable outcomes, ranging from substantial reductions in viable cell counts after hygienisation to only minor or negligible effects, depending on treatment intensity and target taxa (van Looveren et al. 2021; Praeg and Klammsteiner 2024; De Volder et al. 2025).

One of the most promising species for insect farming is the black soldier fly (BSF). Its developmental time is short and BSFL have a high nutritional value that is suitable for feed application in aquaculture, pig and poultry farming (Veldkamp and van Niekerk 2019; Röthig et al. 2023). BSFL can be reared on challenging substrates, including industrial side streams from food processing, which is crucial to produce protein competitive to fishmeal and soybean prices (Klüber et al. 2024; Cattaneo et al. 2024; Rawski et al. 2020; Heuel et al. 2022; Siddiqui et al. 2022; Bajra et al. 2025). For example, there were around 11.6 million tons of potatoes, 4.2 million tons of rapeseed and turnip rape, and 0.8 million tons of apples produced in the fiscal year 2023/2024 in Germany, with an average of 17% food waste from industrial primary production and processing (BMEL 2025a, 2025b, 2025c; BMELH 2025). While the effects of these industrial side streams on BSFL development and gut microbiota have been evaluated in detail, it remains to be determined how they affect insect frass and its fertilising capacities. Insects could be produced more economically by using industrial side streams, which may be bolstered by a thorough characterisation of the valuable add‐on products including frass (Gurusinga et al. 2026; Basri et al. 2022; Beesigamukama et al. 2022).

To this end, we characterised five different BSFL feeds, the respective frass, and the frass‐associated bacterial and fungal communities from heat treated and untreated frass. We employed 16S rRNA gene and ITS2 amplicon sequencing to characterise the frass‐associated microbial communities and linked them to the insect feed composition and heat treatments. We further determined the total colony forming unit (CFU) count of bacteria and fungi, as well as the count of coliforms and *Lactobacillales* to evaluate whether the heat treatment affected the microbial load. Furthermore, we isolated frass‐associated bacteria and identified them via Sanger sequencing of the 16S rRNA gene.

We hypothesise that feed type is the dominant driver of frass‐associated bacterial and fungal community composition, while thermal hygienisation primarily affects microbial viability. We further hypothesise that hygienisation will have smaller effects on community structure inferred from amplicon sequencing than on culture‐based viable counts. Finally, we hypothesise that bacteria and fungi differ in their response to hygienisation, resulting in taxon‐specific changes in viability and, potentially, detectable shifts in community composition.

## 2 Materials & Methods

### 2.1 Rearing and Experimental Set‐Up

BSF originated from Bio. S Biogas GmbH (Grimma, Germany) and were reared in mesh cages in a greenhouse (Klüber et al. 2020). Egg clutches of 150 mg each (approximately 6000 eggs (Klüber et al. 2024)) were placed in closed plastic boxes (19.5 × 16.5 × 9.5 cm) in triplicate for each of the five feed types (15 boxes in total), sprayed with water, and stored in the dark at 27°C ± 1°C and 65% ± 5% relative humidity until hatching. Temperature and humidity were continuously monitored (Testo 174H, Testo SE & Co., KGaA Titisee‐Neustadt/Germany). After hatching of 50% of an egg clutch, the respective lid was replaced with fine mesh and larvae were fed. Five different feed types were provided, including chicken feed (CF, Golddott Eierglück, Raiffeisen, Germany) as control and four biogenic industrial side streams: apple pomace (AP, Kelterei Müller, Butzbach, Germany), potato peelings (PE) and potato pulp (PP, Fraunhofer IVV, Freising, Germany), and rapeseed cake (RC, Gültsteiner Mühle, Herrenberg, Germany). For each feed type (AP, CF, PE, PP, and RC), the plastic rearing boxes (*n* = 3) were placed inside a larger feed type box, which was closed with a fine mesh. The larger boxes were placed on stainless steel racks inside the climate chamber, at heights ranging from 100–130 cm. CF and RC were sourced dry and stored at room temperature. AP, PE, and PP were sourced wet and frozen and stored at—20°C until processed. CF was ground to 100–1500 μm (Mockmill 200, Wolfgang Mock, Otzberg, Germany), rapeseed cake to < 750 μm (spice mill EGK 200, Rommelsbacher, Dinkelsbühl, Germany), and AP was oven‐dried and ground with the spice mill to 100–1500 μm. Frozen PE and PP were processed into slurries (Thermomix TM6, Vorwerk, Wuppertal, Germany).

During the experiments, larvae were fed *ad libitum* and generously sprayed with water daily. Moisture of the frass was monitored daily using a moisture metre (MC‐7828SOIL, Landtek Instruments Co. Ltd., Guangzhou, China) and adjusted to 70% during the larval feeding phase. The larvae were weighed as soon as handling was safe and throughout the feeding trials (Table S5, Barth et al. 2025; Gurusinga et al. 2026). As soon as 50% of larvae within one experimental batch reached prepupal stage, the respective treatment ended. Treatments lasted 17 days for CF, 25 days for RC, 27 days for PE, 52 days for PP, and 73 days for AP. By the end of the experiment, moisture of the frass reduced from 70% to below 60% through cessation of spraying to facilitate the separation of larvae from frass. Larvae were separated from the frass using an automated sieve (5 to 2 mm mesh, Retsch, Arzberg, Germany). The separated larvae were deactivated at −20°C, and frass was collected separately for each feed type.

### 2.2 Sample Preparation and Analysis

Feed samples were collected at the beginning (15 samples) and frass samples at the end of each feeding trial in triplicates (15 samples). Frass samples were subsampled for hygienisation (1 h at 70°C in an oven, BDA‐15, Beeketal, Rastdorf, Germany). Triplicate aliquots of untreated and hygienised frass (30 frass samples) were used for the analysis of colony forming units and culturing, and the remaining samples (untreated frass, hygienised frass, and feed) were frozen and stored at −20°C until further analysis.

Feed samples were analysed for dry matter (DM), ash, crude protein, crude fibre, and crude fat content in triplicates (Klüber et al. 2024). Briefly, samples were ground in a mortar with liquid nitrogen. Homogenised subsamples were taken for DM analysis. The moisture content of each feed type was measured (M35 Moisture Analyser, Sartorius, Göttingen, Germany) and used to calculate DM. The remaining samples were lyophilised at 0.800 mbar and 20°C (Delta 2–24 LSCplus, Martin Christ Gefriertrocknungsanlagen GmbH, Germany). Ash content was measured by weight difference after incineration (6 h, 550°C) of samples (1 g) in an oven (Model L 9/11, Nabertherm, Lilienthal, Germany). Nitrogen content was determined from the samples (1 g) after Kjeldahl (Kjeldatherm, Königswinter, Germany), supported by automated distillation (Vapodest 500, Gerhardt, Königswinter, Germany) and titration (TitroLine 5000 SI Analytics, Mainz, Germany) to calculate protein content with a conversion factor of 6.25. The crude fibre was analysed following Scharrer‐Kürschner (Matissek et al. 2018) by using 3 g of each sample. Crude fat was determined from lyophilised samples (1 g) through acid digestion, filtration, neutralisation, drying (2 h at 105°C), and automated extraction (Soxtherm System, Gerhardt, Königswinter, Germany) with n‐hexane. For the frass samples, only the moisture content was determined.

### 2.3 DNA Extraction, Amplicon Sequencing, and Data Analyses

DNA from 100 mg frass was extracted using the NucleoSpin Soil kit (Macherey‐Nagel, Düren, Germany) with prior cell wall disruption using a high‐speed benchtop homogeniser (FastPrep24, MP Biomedicals, Solon, OH, USA) twice for 1 min at 6.5 m/s homogenisation speed. Extracted DNA was verified spectrophotometrically on the Eon Microplate Spectrophotometer with Gen5 v2.09 (BioTek, Winooski, VT, USA). After passing quality control PCRs, DNA was sent to LGC Genomics (Berlin, Germany) for library preparation and sequencing. Libraries were constructed using the primers U341F (5′‐CCT AYG GGR BGC ASC AG‐3′) and U806R (5′‐GGA CTA CNN GGG TAT CTA AT‐3′) targeting region V3–V4 of the 16S rRNA gene of bacteria and archaea (Sundberg et al. 2013). To amplify the fungal ITS2 region, primers fITS7 (5′‐GTG ART CAT CGA ATC TTT G‐3′) (Ihrmark et al. 2012) and ITS4 (5′‐TCC TCC GCT TAT TGA TAT GC‐3′) were used (White 1990). Sequencing was performed on an Illumina MiSeq V3 platform targeting 20,000 paired‐end reads per sample with a read length of 300 bp.

Sample demultiplexing, adapter and primer clipping were carried out by LGC Genomics using bcl2fastq v2.20 software (Illumina Inc., San Diego, CA, United States). Reads were analysed using the QIIME2 2024.5 amplicon distribution (Bolyen et al. 2019). ITS2 sequences were trimmed if the synthesised strand reached the second sequencing adapter (read‐through) using the cutadapt plugin (Martin 2011) before further analysis. Only the ITS2 forward reads were maintained for further analyses due to insufficient read overlap. We used the DADA2 plugin (Callahan et al. 2016) for error correction, quality control, filtering of chimeric sequences, and inferring and counting of amplicon sequence variants (ASV) across all samples.

ASV classification was conducted in QIIME. Bacterial and archaeal taxonomic classification was carried out using a self‐trained naïve Bayes classifier on SILVA 138.2 QIIME‐compatible release with 99% sequence identity (Quast et al. 2013). We trimmed the reference sequences to the targeted 16S rRNA gene region (Werner et al. 2012). Of note, no archaeal sequences were identified. Fungal taxonomic classification was carried out using a self‐trained naïve Bayes classifier on UNITE 10 QIIME‐compatible release with 99% sequence identity (Abarenkov et al. 2024). Both taxonomy classifiers were built with the RESCRIPt plugin including a patch corresponding to commit 839598b (Robeson et al. 2021). For ASV classification, the minimum confidence was set to 0.7 for 16S and 0.94 for ITS2 (Bokulich et al. 2018). The family assignments of 19 bacterial genera (41 ASVs) have been manually renamed to their validly published families at the time of writing according to the List of Prokaryotic names with Standing in Nomenclature (LPSN) (Parte et al. 2020) (Table S4). All unclassified ASVs or those classified to the kingdom level only were further assessed in a BLASTN v2.16.0 search (word size 11, E‐value 0.05, max target seqs 10) against the preformatted nt BLAST database from NCBI (Aug 7, 2024 2:22 AM, downloaded August 13, 2024) (Altschul et al. 1990; Sayers et al. 2022). Results underwent a manual selection process: (1) non‐target ASVs were discarded (i.e., non‐target kingdom, insect, plant, chloroplast, or mitochondrion), (2) if sequence identity > 80% with an alignment > 140 bp, results were updated to the according target kingdom, with confidence set to −1, (3) all others were flagged as unassigned and discarded. Unwanted sequences (mitochondrial, chloroplast, unclassified, or non‐target kingdom) were removed from the datasets. For the abundance figures, taxonomy was re‐labelled with N/A for NA, unidentified, uncultured, metagenom, and Incertae_Sedis.

For calculating alpha and beta diversity metrics (species richness, Pielou's evenness, Shannon index, and unweighted UniFrac), 16S data were rarefied to 16,492 contigs and ITS2 data to 11,783 reads per sample within the QIIME pipeline (diversity plugin with core‐metrics‐phylogenetic command). To test for differences in alpha diversity between feed types (AP, CF, PE, RC, PP) and heat treatment (untreated vs. hygienised), we used base R (v4.2.2) for statistical testing. Upon assumption verification, alpha diversity measures were assessed by two‐way analysis of variance (ANOVA) followed by pairwise comparisons using Bonferroni in OriginPro 2024 (SR1 10.1.0.178). Beta diversity metrics obtained from the QIIME pipeline were used to identify further differences in microbial community composition based on feed types and treatments. We calculated a Principal Coordinate Analysis (PCoA) based on an unweighted UniFrac to visualise the data. Next, we statistically compared feed types and treatments for significant differences using permutational analysis of variance (PERMANOVA, 999 permutations, seed 123; adonis2, Table S3.3, Table S3.6) in the R package vegan (Oksanen et al. 2024).

### 2.4 Culture‐Dependent Analyses of Frass‐Associated Bacteria

Amplicon sequencing cannot differentiate between DNA from viable and non‐viable sources. To identify and culture viable bacteria and fungi and to further assess the effect of heat treatment on these, subsamples from untreated and hygienised frass were taken. For each feeding trial (AP, CF, PE, PP, RC) 1 g subsamples were taken from each of the triplicate boxes for each heat treatment (30 subsamples in total). The subsamples from each triplicate box were pooled, resulting in a treated and an untreated 3 g sample for each frass type (10 samples in total). For the next steps, 1:10 serial dilutions (six dilutions from 10^−1^ to 10^−6^) of each frass were prepared in peptone water (1.0 g peptone in 1 L water, pH 7.0 (Bast 2014) at a volume of 10 mL) and carefully homogenised by handshaking with two sterile glass beads. From each prepared dilution, 0.2 mL was plated on Petri dishes (94 × 16 mm) in duplicates. The dishes contained either general or selective media, including Casein‐Peptone Soymeal‐Peptone Agar (Carl Roth, Karlsruhe, Germany), Chromogenic Coliform Agar (Merck, Darmstadt, Germany), De Man–Rogosa–Sharpe Agar (Carl Roth, Karlsruhe, Germany), and Yeast Glucose Chloramphenicol Agar (Carl Roth, Karlsruhe, Germany). The Anaerobic Basal Broth (Oxoid Ltd., Basingstoke, United Kingdom) and Jensen's Media (HiMedia 2021) were used only for differentiation without CFUs. The plates were incubated at 27°C and colonies counted after 24 and 72 h. Fungal CFU were enumerated on YGC after 72 h; where plates were countable, colonies were classified as yeast‐like or filamentous based on morphology, whereas confluent growth at low dilutions precluded reliable categorisation. Mean CFU values were calculated from plates with countable colonies (according to ISO 4833‐1:2013/Amd 1 2022) and normalised to the dry matter. Dry matter (DM) was determined on 1 g of the corresponding untreated frass sample (M35 Moisture Analyser, Sartorius, Göttingen, Germany). After CFU counting, up to four bacterial colonies per feed and medium type were taken and further isolated to obtain optically pure cultures. The CFU data was statistically tested for differences between heat treatments, feed types, and culture media by PERMANOVA (adonis2 function in the R package vegan, 9999 permutations, seed 20) (Table S3.7).

For taxonomical identification of the isolates, template DNA was extracted from the cultures. Firstly, DNA was extracted by digestion in 0.2% sodium dodecyl sulfate (SDS) or dimethyl sulfoxide (DMSO) at 95°C for 10 min. The samples were incubated at 95°C for 10 min in a heating block and then vortexed and centrifuged at 2900 g for 2 min. For SDS samples, 2 μL were taken immediately afterwards and diluted with 18 μL nuclease‐free water. For samples that did not yield suitable template DNA (insufficient quantity or quality), we homogenised the sample in a TissueLyser (QIAGEN, Hilden, Germany) with glass beads at 30 Hz for 1 min. Samples were then centrifuged at 2900 g for 2 min, incubated at 70°C, centrifuged again, and the DNA extracted from the supernatant. Template DNA was amplified using the 27F and 1492R primers (Lane 1991). Amplified DNA was Sanger‐sequenced at Microsynth Seqlab (Göttingen, Germany). The sequence data were quality controlled and trimmed in the CLC Main Workbench v20.0.4 (QIAGEN, Netherlands) and a BLAST search (*E*‐value 10.0; word size 16; match 1; mismatch −2; gap costs for existence 5 and extension 2; max target seqs 100) against the NCBI core nucleotide database was performed for taxonomic classification. All sequences were classified to the lowest feasible taxonomic rank, but results are reported at the family level to ensure a meaningful comparison between culture‐independent (amplicon sequencing) and culture‐dependent (Sanger sequencing of isolates) datasets. Isolates classified to the genus *Lysinibacillus* were assigned to the family *Caryophanaceae* according to the nomenclature of LPSN (Table S4).

For further analysis, CD‐HIT‐EST v4.8.1 (Fu et al. 2012) was used to cluster all Sanger sequences (word size 10, length difference cutoff 90%) at sequence identities of 99% and 100%. Cluster membership was used to compare isolate sequences across frass types (as defined by the rearing feed) and heat‐treatment conditions (untreated vs. hygienised) independent of taxonomic classification. For 100% identity, we subsequently applied VSEARCH v2.30.1 (Rognes et al. 2016) for pairwise alignment of all ASVs against the clustered Sanger sequences, utilising the—usearch_global option (sequence identity 100%, maxgaps 0, searching the complete database). For further analysis, ASVs were categorised as either matched or unmatched against our Sanger sequences and combined with their corresponding relative abundances across all samples.

## 3 Results

We assessed microbial load and community composition in BSFL frass as a function of feed type and thermal hygienisation. Bacterial and fungal communities were analysed in frass derived from apple pomace (AP), chicken feed (CF), potato peelings (PE), potato pulp (PP), and rapeseed cake (RC) under both untreated and hygienised conditions. Throughout the manuscript, we refer to each frass type by the corresponding rearing feed (AP, CF, PE, PP, RC).

The control feed and industrial side streams were characterised for protein, fat, ash, dry matter, and fibre contents to contextualise frass microbial patterns across feed types.

### 3.1 Composition of BSFL Feeds

The control feed CF and all four biogenic side streams used as BSFL feed differed markedly in their composition (Table 1). Compared to all other feeds, CF contained the highest proportion of dry matter (DM) (91.44% ± 0.1%) and ash (12.94% ± 0.94% DM) but was lowest in crude fibre (9.9% ± 2.7% DM). AP included the lowest content in dry matter (22.94% ± 2.55%) and ash (1.24% ± 0.24% DM) and the highest proportion of crude fibre (34.3% ± 6.6% DM) compared to all other feeds. PE contained the lowest amount of raw protein (7.54% ± 0.81% DM) and PP the lowest proportion of crude fat (0.15% ± 0.20% DM); both components were measured highest in RC (20.93% ± 0.16% DM and 17.16% ± 0.36% DM, respectively). The prominent differences in feed composition provide a base to investigate the effects of BSFL feed composition on microbial frass communities.

**TABLE 1:** Composition of insect feed types. Displayed are chicken feed (CF) as the control diet and the biogenic side streams apple pomace (AP), potato peelings (PE), potato pulp (PP), and rapeseed cake (RC) in triplicate measurements with standard deviation. CF, PP (Klüber et al. 2024), and PE (Barth et al. 2025) have been previously published and are included for comparison. DM = dry matter.

Feed type	Dry matter (%)	Raw protein (% DM)	Crude fat (% DM)	Ash (% DM)	Crude fibre (% DM)
Apple pomace (AP)	22.94 ± 2.6	5.16 ± 0.2	1.67 ± 0.1	1.24 ± 0.2	34.3 ± 6.6
Chicken feed (CF)	91.44 ± 0.1	18.20 ± 0.4	2.50 ± 0.1	12.94 ± 0.9	9.9 ± 2.7
Potato peelings (PE)	18.12 ± 3.2	7.54 ± 0.8	0.43 ± 0.1	2.75 ± 0.1	15.5 ± 1.4
Potato pulp (PP)	24.16 ± 1.0	7.61 ± 0.03	0.2 ± 0.2	5.93 ± 0.3	10.8 ± 3.4
Rapeseed cake (RC)	94.07 ± 0.1	20.93 ± 0.2	17.16 ± 0.4	6.26 ± 0.1	12.4 ± 2.2

### 3.2 Characterisation of Bacterial Communities

We characterised BSFL frass‐associated bacterial communities to identify differences related to feed type and heat treatment (hygienisation) by 16S rRNA gene amplicon sequencing. Following DNA extraction, amplification, library preparation, and sequencing, we gained 958,519 read pairs across 30 samples, consisting of triplicates from untreated and hygienised frass samples from each of the five feed types (AP, CF, PE, PP, RC). After trimming, denoising, chimaera detection, and removal of unwanted taxa, the average number of read pairs that were merged into contigs per sample was 22,351, ranging from 16,492–30,195 (Figure S1A). In total, we identified 463 distinct bacterial ASVs with an average length of 419 bp across the remaining 670,527 contigs.

**FIGURE 1:**
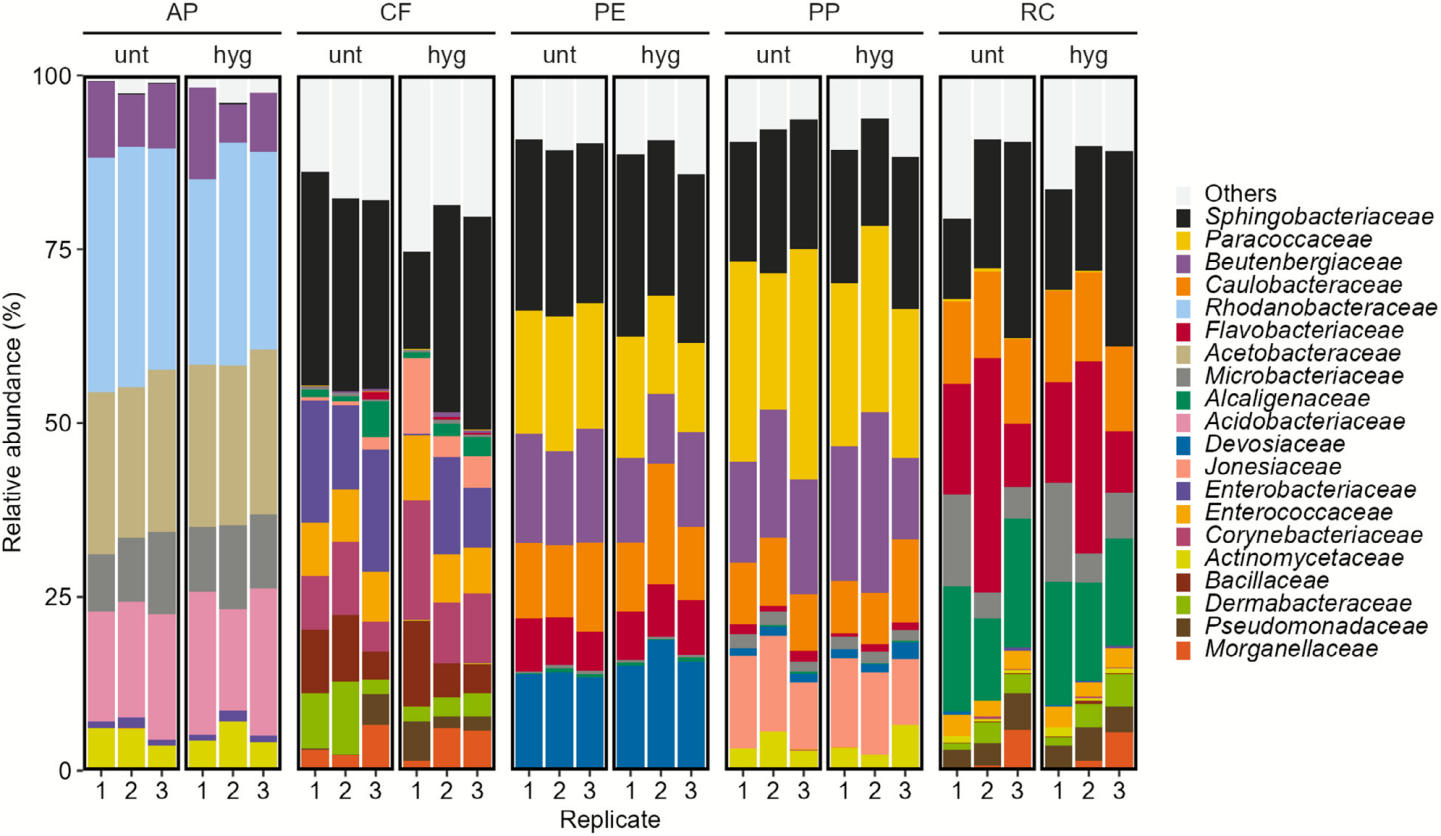
Bacterial community composition in *Hermetia illucens* frass across feed types and heat treatments. Stacked column plot depicting relative bacterial abundances on the taxonomic level family for the different feeds (AP = apple pomace, CF = chicken feed, PP = potato pulp, PE = potato peelings, RC = rapeseed cake) in untreated (unt) and hygienised (hyg) frass samples. The 20 most abundant families across all samples are displayed, and all remaining taxa (*n* = 54) were grouped as “Others”. The relative abundances are based on unrarefied data.

We first characterised bacterial communities at the taxonomic level family to assess community composition and differences between insect feed types and frass heat treatment (Figure 1, Table S1, Figure S2). Bacterial communities differed notably between feed types, with PE and PP being most similar, while treatments had no consistent effect on the bacterial compositions. *Sphingobacteriaceae* (22.3%), mainly the genus *Sphingobacterium*, dominated across all samples except for AP (0.1%), where its relative abundance was low. The relative abundance of *Paracoccaceae* was high in PE (16.6%) and PP (25.5%) and low in CF (0.05%) and RC (0.2%). *Beutenbergiaceae* were mainly found in PE (13.6%), PP (17.8%), and AP (9.2%), and at a low relative abundance in CF (0.3%). Similarly, *Caulobacteraceae* were relatively abundant in PE (11.9%), PP (8.9%), and RC (12.4%). In contrast, *Rhodanobacteraceae* (31.2%), *Acetobacteraceae* (23.1%), and *Acidobacteriaceae* (17.8%) occurred only in AP. *Enterobacteriaceae* and its predominant genus *Klebsiella* showed a high relative abundance in CF (11.6%), a low relative abundance in AP (1.1%), PE (0.1%), and RC (0.15%), and were absent in PP. *Flavobacteriaceae* were more common in RC (18.3%) and PE (7.1%) compared to CF (0.3%) and PP (1.1%) and not detected in AP. *Bacillaceae* were common in CF (7.4%) and present in RC (0.2%) and in one sample of untreated PP (< 0.1%). *Alcaligenaceae* were much more abundant in RC (16.0%) compared to CF (2.1%), PE (0.37%), and PP (0.15%), and one hygienised sample of AP (< 0.1%). Only *Microbacteriaceae* were present in all 30 samples, although their relative abundance was markedly higher in AP (10.2%) and in RC (7.8%) than in CF, PE (each 0.4%), and PP (1.7%). Overall, the bacterial community compositions reflect the uneven and inconsistent distribution of bacterial taxa, particularly between frass from different feed types. Here, the bacterial composition in BSFL frass revealed distinct patterns on the family level reflecting feed type correlated differences but lacking an apparent heat treatment structure.

To further assess differences between feed types and treatment effects, we calculated alpha diversity metrics including species richness, Pielou's evenness, and Shannon index for all samples (Table 2). Across all feed types, AP consistently exhibited the lowest values in species richness (42.67 ± 8.08), Pielou's evenness (0.61 ± 0.01), and Shannon index (3.29 ± 0.13). CF displayed consistently the highest values (100 ± 2.08, 0.80 ± 0.02, and 5.30 ± 0.13, respectively) compared to all other feed types (Table 2). Statistical testing revealed significant differences between feed types (p_ANOVA_ < 0.0001), whereas heat‐treated samples did not differ significantly (p_ANOVA_ = 0.2426) (Table S3.1). Pairwise tests showed significant differences between the bacterial communities in CF frass and the frass samples from AP, PP, and RC, and between AP and the samples from PE and RC (Table S3.2) while all other samples were not significantly different from each other.

**TABLE 2:** Overview of alpha diversity metrics based on rarefied data in bacterial communities associated with untreated and hygienised *H. illucens* frass originating from five insect feeds (AP, CF, PP, PE, and RC). Displayed are averages (*n* = 3) with standard deviation.

Feed type	Number of reads		Species richness		Pielou's evenness		Shannon index	
	Unt	Hyg	Unt	Hyg	Unt	Hyg	Unt	Hyg
AP	22,455 ± 3179	19,043 ± 1982	42.67 ± 8.08	42.67 ± 5.51	0.61 ± 0.01	0.65 ± 0.02	3.29 ± 0.13	3.49 ± 0.03
CF	21,578 ± 2673	21,436 ± 3394	84.00 ± 15.13	100.00 ± 2.08	0.76 ± 0.04	0.80 ± 0.02	4.87 ± 0.45	5.30 ± 0.13
PE	20,972 ± 3908	23,549 ± 3305	77.67 ± 14.43	88.00 ± 6.93	0.72 ± 0.02	0.72 ± 0.01	4.51 ± 0.14	4.64 ± 0.14
PP	21,205 ± 520	25,013 ± 1621	57.00 ± 6.93	61.00 ± 2.64	0.67 ± 0.03	0.66 ± 0.05	3.90 ± 0.07	3.92 ± 0.29
RC	24,512 ± 5146	23,746 ± 5573	69.67 ± 11.59	72.00 ± 14.84	0.71 ± 0.05	0.72 ± 0.06	4.34 ± 0.35	4.44 ± 0.26

**FIGURE 2:**
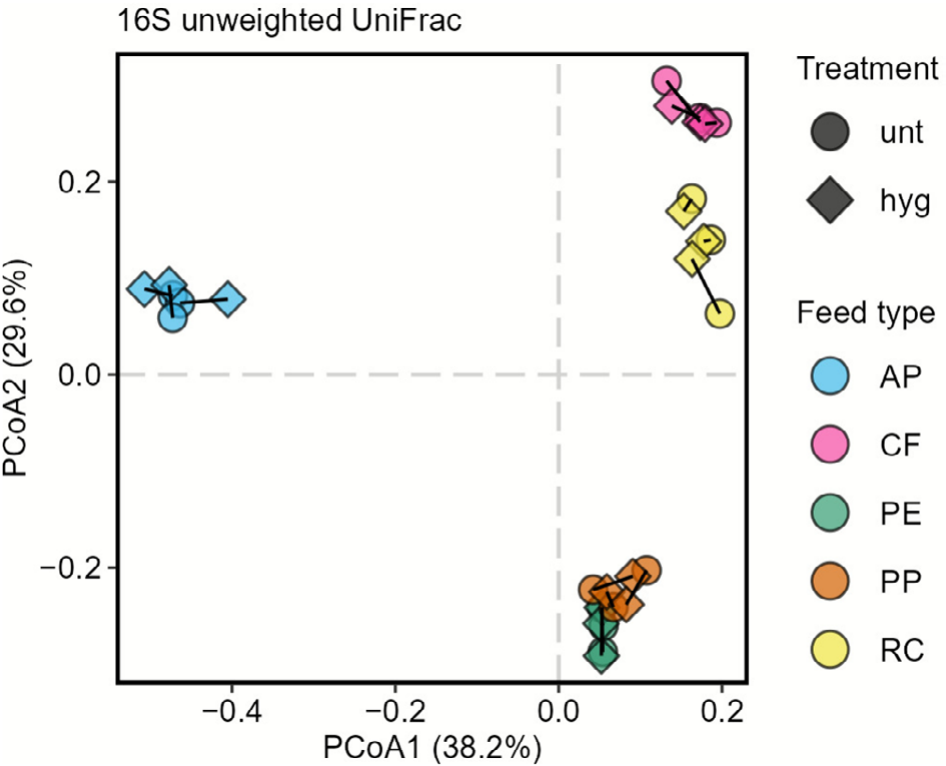
Principal Coordinates Analysis based on unweighted UniFrac distance matrix visualising differences in bacterial community structure of untreated (unt) and hygienised (hyg) *H. illucens* frass from five feed types (AP, CF, PP, PE, RC). Colours denote feed type; shapes denote treatment. Untreated and hygienised samples from the same origin are connected by lines. Data shown are based on rarefied data.

Next, we visualised differences between the bacterial communities in an ordination plot based on an unweighted UniFrac distance matrix (Figure 2). The Principal Coordinates Analysis (PCoA) showed that samples from the same feed type generally clustered closely together, indicating a high similarity in their bacterial communities. By comparison, samples from different feed types clustered away from each other, except for PE and PP samples, which clustered closely together. Notably, none of the feed‐type clusters showed a clear separation between untreated and hygienised samples.

We next statistically evaluated differences between feed types and treatments. We identified feed type to highly significantly (p_PERMANOVA_ = 0.001) affect bacterial community compositions, explaining 85.0% of the variation. However, heat treatment did not significantly affect community composition (p_PERMANOVA_ = 0.623), explaining only 0.4% of the variation (Table S3.3). These data highlight the dominant role of the feed type in shaping frass‐associated bacterial community structure.

Taken together and based on bacterial community composition assessed on the taxonomic level family, alpha diversity indices, PCoA, and PERMANOVA results, frass‐associated bacterial communities were driven by the insects feed type and, by comparison, little affected by the frass' heat treatment. However, it is important to acknowledge that DNA amplification‐based analyses do not discern DNA material from viable or dead microbes and are therefore only of limited use to evaluate the hygienisation effect.

### 3.3 Characterisation of Fungal Frass Communities

We further examined frass‐associated fungal communities, which are also involved in insect digestion, particularly with nutritionally challenging materials such as fibre. Analogous to the bacterial community assessments described above, we employed ITS2 region amplicon sequencing to identify fungal community differences driven by feed types and heat treatment. DNA extraction and amplification, library preparation and sequencing yielded 768,523 read pairs for 30 frass samples from untreated and hygienised treatments for each feed type (AP, CF, PE, PP, and RC) which were sampled in triplicates each. Read pairs were not sufficiently overlapping, and only forward reads were used for the analyses. After quality trimming, denoising, chimaera detection, and removal of 18 non‐target ASVs, a total of 436,759 single‐end reads remained in the dataset. The number of cleaned reads per sample ranged from 11,783 to 46,592 with a mean of 23,449, indicating sufficient sequencing depth for all samples (Figure S1B). In total, 122 distinct fungal ASVs across all 30 samples were classified. ASVs had an average size of 254 bp with a minimum of 128 bp and a maximum of 273 bp. The frass samples contained a distinctly lower fungal richness compared to bacteria, ranging between 3 and 28 distinct fungal ASVs per sample.

**FIGURE 3:**
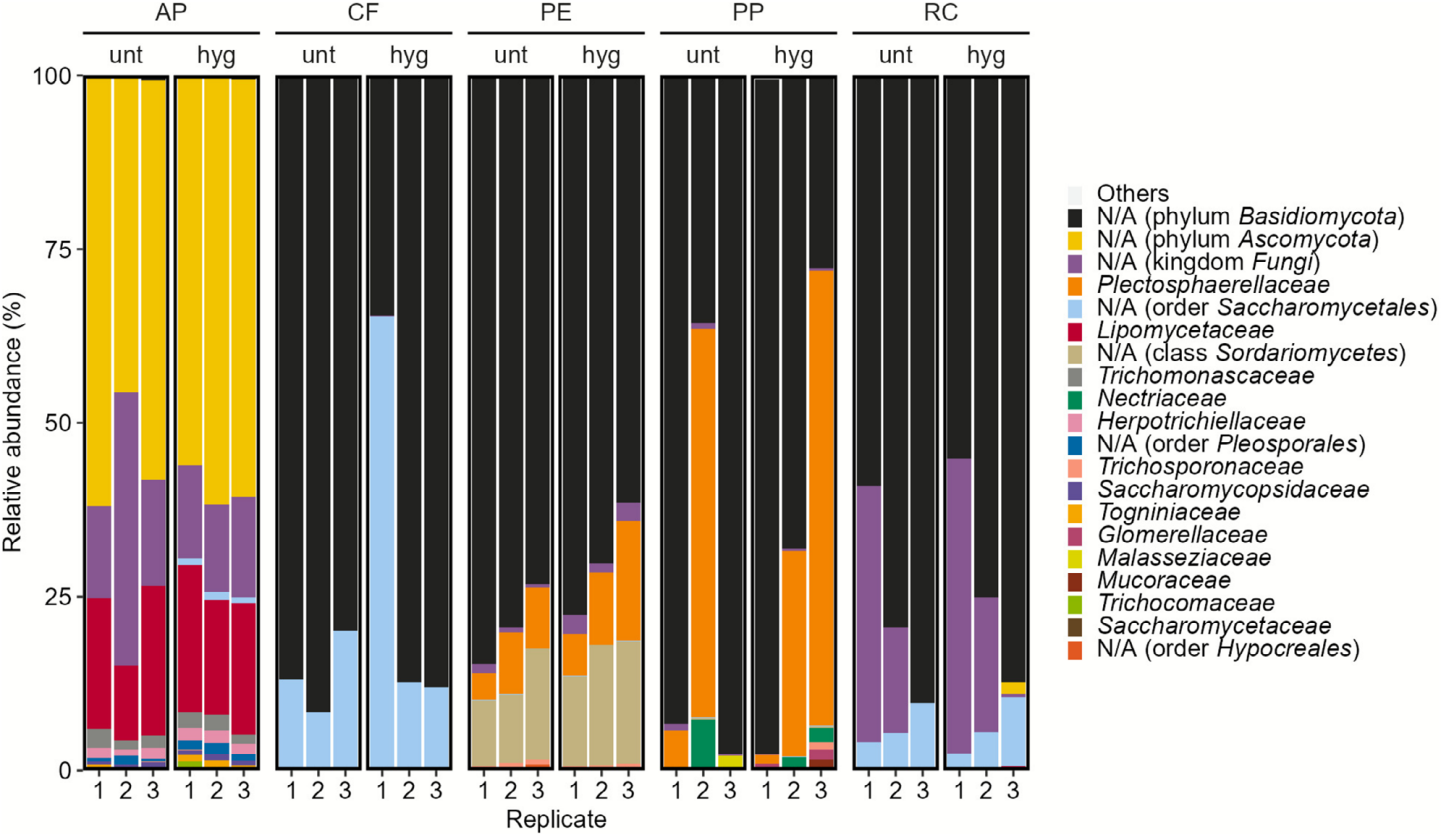
Fungal community composition in *H. illucens* frass across feeds and heat treatments. Stacked column plot depicting relative fungal abundances at the phylogenetic level of family for the different feeds (AP, CF, PP, PE, RC) in untreated (unt) and hygienised (hyg) frass samples. The 20 most abundant families across all samples are displayed. All remaining fungal families (*n* = 22) were grouped into “Others”. The relative abundances are based on unrarefied data.

We next assessed fungal community composition on the taxonomic level family. Across all samples, fungal communities were markedly different between different feed types whereas heat treatment did not seem to have an effect (Figure 3, Table S1, Figure S3). Five ASVs within *Basidiomycota* could not be classified to the family level and were assigned only to the phylum. They were dominant (59.7%) across most of the samples, even though they were almost absent in the AP samples (0.2%). AP samples were dominated by *Ascomycota* (81.7%) with two abundant ASVs unclassified at family level (56.9%), which were negligible or not detected in all other samples. Further highlighting stark differences between frass from feed types, *Saccharomycetales* in AP (18.0%) were dominated by the genus *Dipodascopsis* (18.0%), while in CF (21.9%) and RC (6.3%) the genus *Diutina* (CF 21.9%, RC 6.2%) was most abundant. However, *Saccharomycetales* were also found in PE (< 0.1%) and PP (0.2%) and are the only fungal order detected in all 30 samples (9.9%). Overall, the different feed types appeared to have a pronounced effect on the fungal frass‐associated communities whereas heat treatment does not have a major impact on the communities based on amplicon sequencing data.

We further calculated fungal alpha diversity parameters including species richness, Pielou's evenness, and Shannon index based on rarefied data to detail the influence of feed type and heat treatment on fungal frass communities (Table 3). Contrasting the trends for bacterial communities, AP showed the highest and CF the lowest values for richness (27.33 ± 0.58 vs. 4.67 ± 1.53) and Shannon index (2.16 ± 0.11 vs. 1.15 ± 0.06). Evenness was highest in RC (0.67 ± 0.16) and lowest in PP (0.35 ± 0.13). Statistical testing confirmed significant differences for feed type (p_ANOVA_ = 0.0021) while hygienisation treatment had no significant effect (p_ANOVA_ = 0.3753) on the fungal frass communities (Table S3.4, Table S3.5).

**TABLE 3:** Alpha diversity measures based on rarefied data of *H. illucens* frass‐associated fungal communities. Data from ITS2 region amplicon sequencing of untreated and hygienised *H. illucens* frass samples from five different feed types (AP, CF, PP, PE, RC). Shown are averages (*n* = 3) with standard deviation.

Feed type	Number of reads		Species richness		Pielou's evenness		Shannon index	
	Unt	Hyg	Unt	Hyg	Unt	Hyg	Unt	Hyg
AP	19,561 ± 135	17,782 ± 5344	21.33 ± 3.00	27.33 ± 0.58	0.46 ± 0.02	0.45 ± 0.02	2.01 ± 0.03	2.16 ± 0.11
CF	28,713 ± 3562	24,654 ± 11,978	4.67 ± 1.53	5.00 ± 1.00	0.66 ± 0.16	0.51 ± 0.05	1.37 ± 0.08	1.15 ± 0.06
PE	16,401 ± 16,25	19,490 ± 6220	11.67 ± 3.06	10.67 ± 0.58	0.43 ± 0.07	0.52 ± 0.06	1.49 ± 0.15	1.77 ± 0.20
PP	23,013 ± 8327	30,886 ± 13,607	13.00 ± 4.36	15.67 ± 2.52	0.35 ± 0.09	0.35 ± 0.13	1.30 ± 0.50	1.38 ± 0.57
RC	31,509 ± 10,754	22,483 ± 4974	4.67 ± 1.53	7.33 ± 3.21	0.67 ± 0.16	0.56 ± 0.16	1.41 ± 0.27	1.51 ± 0.17

**FIGURE 4:**
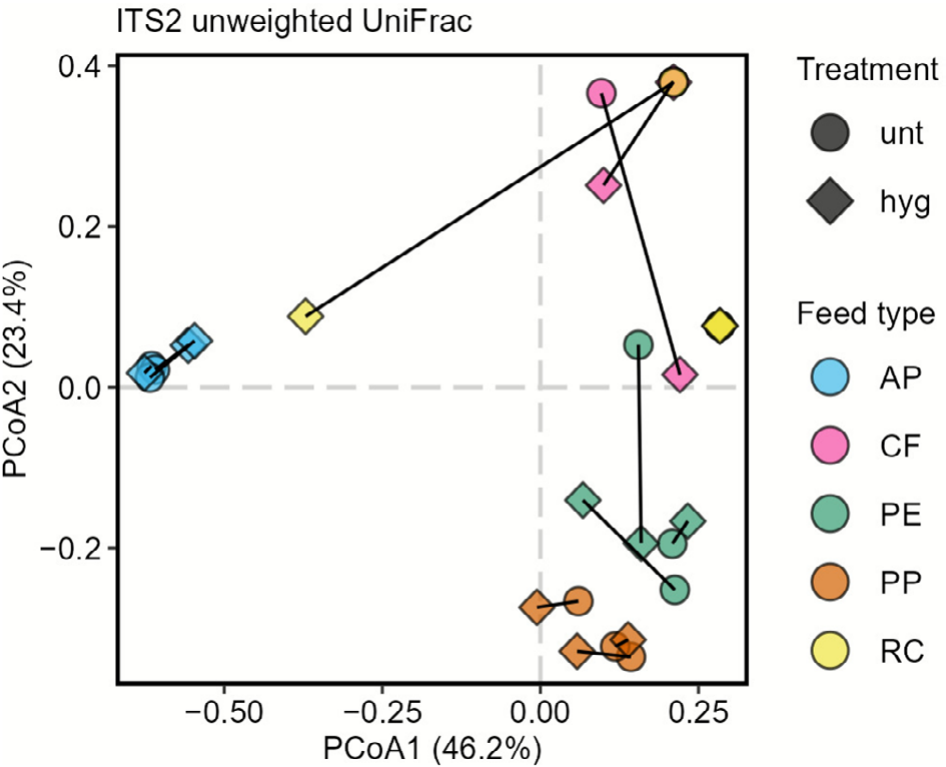
PCoA on unweighted UniFrac distance matrix representing fungal community structure of untreated (unt) and hygienised (hyg) *H. illucens* frass from five feed types (AP, CF, PP, PE, RC). Colours denote feed type and shapes denote treatment. Untreated and hygienised samples from the same origin are connected by lines. Data shown based on rarefied data.

We next compared fungal community composition between samples to characterise differences based on feed type and heat treatment. Upon visualisation in a PCoA, fungal communities generally clustered according to feed types (Figure 4). Specifically, AP, PE and PP samples formed close clusters each, away from the other samples. By comparison, CF and RC displayed a broader scattering. In contrast to the feed type, the heat treatment did not seem to affect the fungal community composition as there was no discernible separation between the according samples. These observations were confirmed by statistical assessment (Table S3.6). PERMANOVA analysis identified feed types to significantly affect community structure, accounting for 68.5% of the variation (p_PERMANOVA_ = 0.001). In contrast, the hygienisation treatment did not significantly influence community composition, explaining only 1.8% of the variation (p_PERMANOVA_ = 0.191). Overall, amplicon‐based data showed that feed types drive microbial (bacterial and fungal) community composition of BSFL frass, whereas the heat treatment seems to have no discernable impact on the community compositions.

### 3.4 Culture‐Dependent Analysis of Frass‐Associated Microbial Members

We next verified the effect of feed type and particularly heat treatments on the viable portion of frass‐associated microbial members using culture‐dependent analyses. We counted CFUs from 80 samples on four selective and non‐selective media across all frass types (5 frass types, 2 heat treatments, duplicates for each of the 4 media) (Table 4). Feed type affected the bacterial counts strongly, which ranged for aerobic bacteria between 5.12 × 10^3^ ± 1.45 × 10^3^ (hygienised PE) and 7.17 × 10^8^ ± 5.42 × 10^8^ (untreated CF), for coliforms between 7.73 × 10^2^ ± 1.07 × 10^3^ (hygienised PE) and 2.71 × 10^7^ ± 2.76 × 10^6^ (untreated CF), for *Lactobacillales* between 4.68 × 10^3^ ± 3.87 × 10^3^ (hygienised PE) and 1.32 × 10^8^ ± 1.20 × 10^7^ (hygienised RC), and for fungi between 1.41 × 10^3^ ± 5.44 × 10^2^ (hygienised PE) and 1.45 × 10^8^ ± 1.77 × 10^7^ (untreated AP).

**TABLE 4:** Overview of viable microbial colony counts from frass. Counts are mean CFU ± SD (*n* = 2) obtained from plates with countable colonies (according to ISO 4833–1:2013/Amd 1 2022). Fungal CFU were enumerated on YGC after 72 h; yeast‐like and filamentous morphotypes were recorded when distinguishable. “n.d.” indicates not detected on countable plates under these conditions; confluent growth at lower dilutions could not be enumerated.

					Fungi	
Feed type	Treatment	Aerobic bacteria	Coliforms	*Lactobacillales*	Filamentous	Yeast like
AP	unt	2.88 × 10^7^ ± 2.33 × 10^6^	2.33 × 10^7^ ± 2.12 × 10^5^	5.50 × 10^7^ ± 2.60 × 10^7^	n.d.	1.45 x 10^8^ ± 1.77 × 10^7^
	hyg	3.26 x 10^6^ ± 2.12 × 10^5^	7.16 x 10^5^ ± 1.31 × 10^5^	1.50 x 10^7^ ± 9.90 × 10^5^	n.d.	1.60 × 10^7^ ± 1.84 × 10^6^
CF	unt	7.17 × 10^8^ ± 5.42 × 10^8^	2.71 × 10^7^ ± 2.76 × 10^6^	9.24 x 10^6^ ± 1.36 × 10^6^	1.45 × 10^4^ ± 2.03 × 10^4^	3.63 × 10^4^ ± 1.03 × 10^4^
	hyg	4.06 × 10^8^ ± 4.10 × 10^7^	9.43 × 10^6^ ± 1.13 × 10^7^	2.36 × 10^7^ ± 2.33 × 10^6^	4.35 × 10^4^ ± 0	5.08 × 10^4^ ± 1.03 × 10^4^
PE	unt	1.83 × 10^6^ ± 3.11 × 10^5^	1.27 × 10^6^ ± 0	5.76 × 10^5^ ± 5.61 × 10^5^	5.24 × 10^4^ ± 5.42 × 10^3^	4.09 × 10^4^ ±7.23 × 10^3^
	hyg	5.12 × 10^3^ ± 1.45 × 10^3^	7.73 × 10^2^ ± 1.07 × 10^3^	4.68 × 10^3^ ± 3.87 × 10^3^	6.39 × 10^2^ ± 5.42 × 10^2^	7.67 × 10^2^ ± 0
PP	unt	2.64 × 10^6^ ± 2.90 × 10^5^	3.75 × 10^5^ ± 1.29 × 10^5^	1.69 × 10^5^ ± 4.24 × 10^3^	1.84 × 10^5^ ± 2.87 × 10^4^	3.04 × 10^3^ ± 1.43 × 10^3^
	hyg	1.06 × 10^7^ ± 1.46 × 10^6^	1.98 × 10^5^ ± 3.58 × 10^4^	1.37 × 10^6^ ± 3.25 × 10^5^	1.00 × 10^6^ ± 1.16 × 10^6^	n.d.
RC	unt	3.55 × 10^8^ ± 5.94 × 10^7^	1.68 × 10^7^ ± 9.90 × 10^5^	3.34 × 10^7^ ± 2.97 × 10^6^	2.99 × 10^7^ ± 2.62 × 10^6^	n.d.
	hyg	2.92 × 10^8^ ± 2.97 × 10^7^	1.87 × 10^7^ ± 4.60 × 10^6^	1.32 × 10^8^ ± 1.20 × 10^7^	2.86 × 10^7^ ± 1.13 × 10^6^	n.d.

Thermal hygienisation appeared to reduce aerobic bacteria and coliforms in most frass types, with differences between 0.09 log in RC and 2.55 log in PE for aerobic bacteria and between 0.28 log in PP and 3.22 log in PE for coliforms. However, this trend was not consistent and CFU counts increased slightly in RC for coliforms and in PP for aerobes. Interestingly, *Lactobacillales* mostly increased in CF from 9.24 × 10^6^ to 2.36 × 10^7^, in PP from 1.69 × 10^5^ to 1.37 × 10^6^, and in RC from 3.34 × 10^7^ to 1.32 × 10^8^. Yet, this trend was not consistent across all samples as *Lactobacillales* decreased in AP from 5.50 × 10^7^ to 1.50 × 10^7^ and in PE from 5.76 × 10^5^ to 4.68 × 10^3^. For total fungi, the treatment effects were heterogenous, with noticeable decreases of 0.96 log in AP and 1.82 log in PE and an increase of 0.73 log in PP. During the differentiation between filamentous and yeast‐like fungi, only yeast‐like fungi could be detected in AP, which were reduced by 0.96 log following heat treatment. In RC, only filamentous fungi were detected, and their change after heat treatment was minimal. In PP, the filamentous fungi increased by 0.74 log after heat treatment, whereas in PE, they decreased by 1.92 log. Similarly, the yeast‐like fungi in PE decreased by 1.73 log, while in PP, they were no longer detectable after heat treatment.

We confirmed the statistical significance of the treatment effect (p_PERMANOVA_ = 0.0371) and post hoc tests revealed no significant differences for both bacteria and fungi (Table S3.7).

**FIGURE 5:**
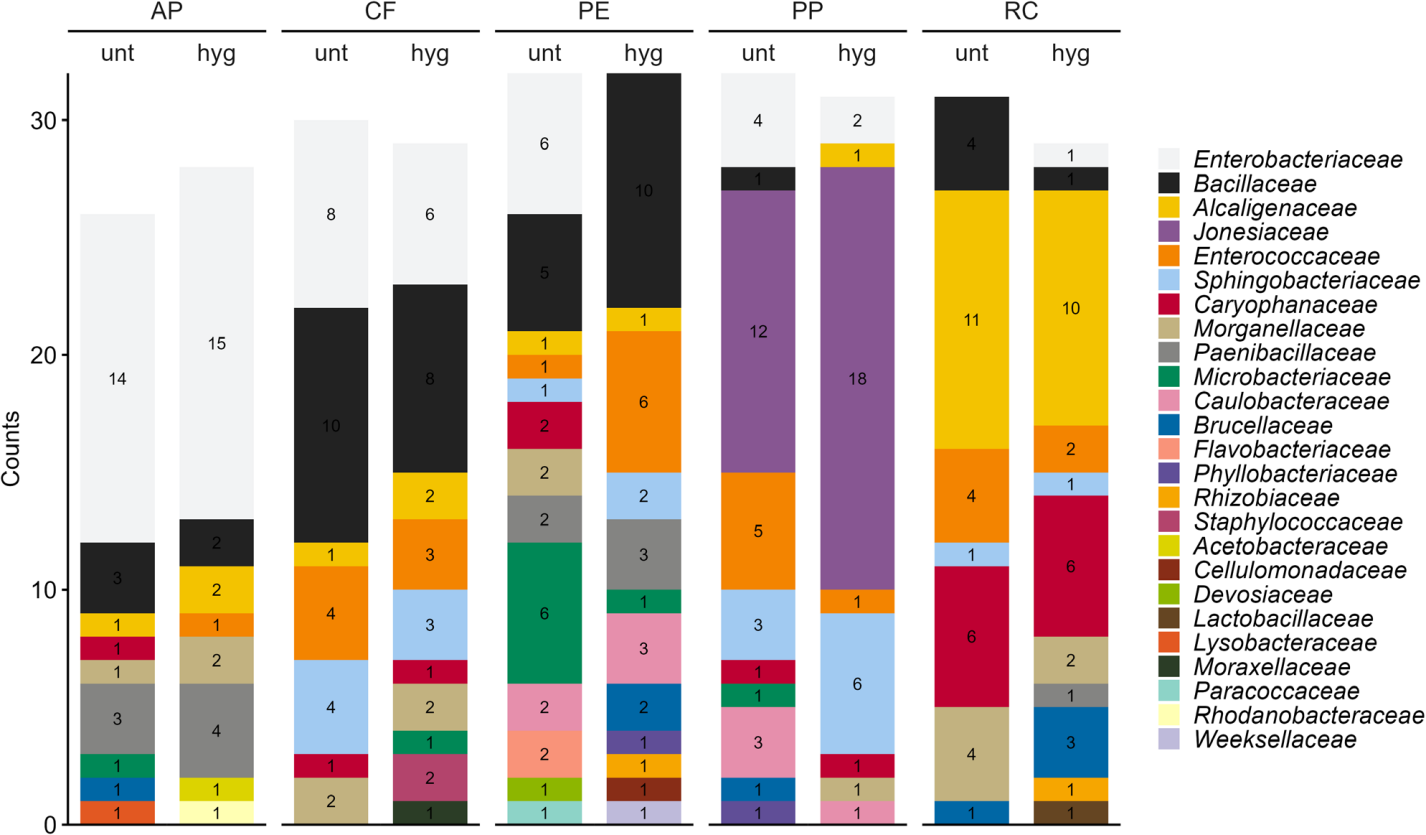
Distribution of cultured and identified bacterial families based on 16S rRNA Sanger sequencing data from untreated (unt) and hygienised (hyg) *H. illucens* frass from five feed types (AP, CF, PP, PE, RC).

Next, we aimed to identify the cultured bacteria. From each sample (*n* = 10) we cultured and classified between 26 and 32 bacteria. Generally, frass samples from the same feed type seemed more similar to each other irrespective of the heat treatment compared to samples from different feed types. Across all samples, *Bacillaceae* were most frequently isolated and present across all frass types and even in all treatments except for hygienised PP. *Enterobacteriaceae* and *Alcaligenaceae* were also consistently found in all frass *types*, with the latter being abundant in RC. Interestingly, the effect of heat treatment seemed to vary between different feed types (Figure 5, Figure S4, Table S2). *Alcaligenaceae* decreased in RC (11 to 9), *Bacillaceae* in CF (8 to 6) and RC (4 to 2), *Enterobacteriaceae* in CF (8 to 6), PE (6 to 0) and PP (4 to 2), and *Enterococcaceae* in CF (4 to 3), PP (5 to 1) and RC (4 to 2). However, the same taxa increased in other feed types. For example, *Bacillaceae* decreased upon heat treatment in the frass of all feed types but PE, where it increased (5 to 10). Similarly, *Enterobacteriaceae* increased in AP (14 to 15) and RC (0 to 1), *Jonesiaceae* in PP (12 to 18) and *Enterococcaceae* in PE (1 to 6). *Paenibacillaceae* increased (3 to 4) in AP and (2 to 3) in PE. Similar to the amplicon sequencing data, feed type generally seemed to affect the classified cultured bacterial taxa; however, there was no clear trend upon heat treatment which might also be affected by an overall comparably low replication rate (26 to 32 isolates per sample type).

To assess whether similar isolate sequences occurred across frass types and heat‐treatment conditions, we examined the distribution of isolated strains across sequence‐based clusters. The top five clusters are shown in Figure S5 and are listed in Table S2. Members of the genus *Klebsiella* with a sequence similarity ≥ 99% form by far the largest cluster, comprising 33 isolates distributed across AP, CF, PE, and PP. The second largest cluster comprises only PP isolates, totaling 23 members of *Populibacterium*. This is followed by *Alcaligenes* (16 isolates) with isolates from RC, CF, and PE; *Enterococcus* (13 isolates) with isolates from RC, PP, PE, and AP; and a *Citrobacter* cluster (7 isolates) with isolates only from PP and PE. These five clusters, out of a total of 137 clusters, together comprise 30.67% of all isolated strains. However, we found no cluster containing isolates from all frass types and both heat‐treatment conditions. We identified two clusters (Numbers 14 and 33) with individuals in at least four frass types, and one cluster (Number 2) with isolates from at least three frass types. All major clusters were present in both untreated and hygenised frass samples, except cluster 36 (*Citrobacter*), which was only found in untreated frass samples (Figure S5).

**FIGURE 6:**
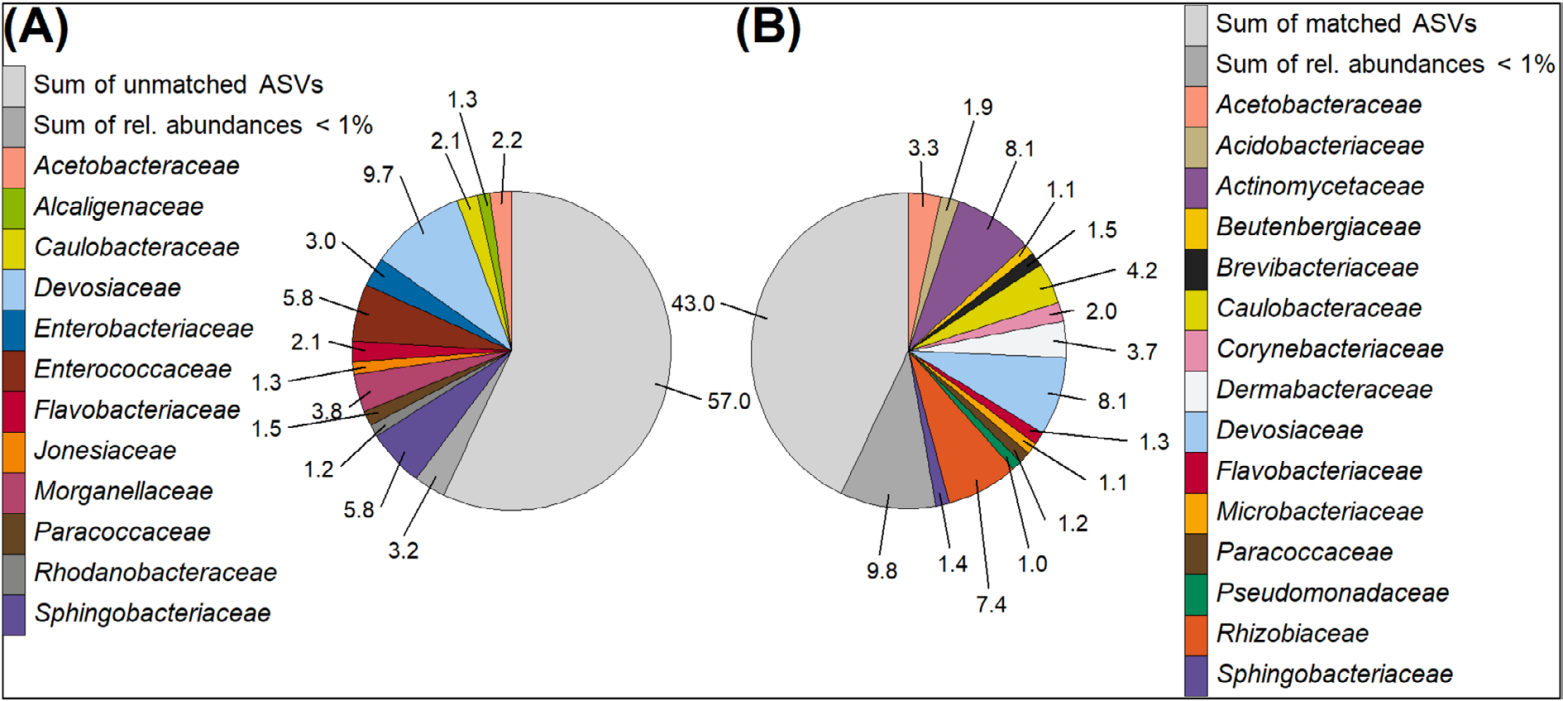
Bacterial composition at family level, depending on ASVs aligned with 100% identity to 293 clustered Sanger sequences. Numbers indicate percent relative abundance. Families with < 1% relative abundance are consolidated. The same families are assigned the same colour. (A) Distribution of 64 ASVs aligned to 222 Sanger sequences (matched, shown in colour); the light grey section represents all unmatched ASVs. (B) Distribution of 399 ASVs not aligned to any of our Sanger sequences (unmatched, shown in colour); the light grey section represents all matched ASVs. Light grey denotes the complementary fraction (unmatched in A; matched in B); dark grey the consolidated fraction of families each contributing < 1% relative abundance.

To assess how effectively our cultivation method captures the dominant microorganisms present in the original samples, we compared the microbial taxa obtained through culture‐dependent techniques with the abundant taxa identified by amplicon sequencing. Across the 16S rRNA amplicon dataset, 74 bacterial families were detected (Figure 1). Cultivation recovered 25 of these families (34%), and we did not detect additional families among isolates beyond those observed in the amplicon dataset. The recovered families included dominant amplicon taxa such as *Sphingobacteriaceae* and *Paracoccaceae* (Figure 1). Aligning all 463 distinct bacterial ASVs against the 293 non‐redundant Sanger sequences using VSEARCH revealed that 64 ASVs matched 222 Sanger sequences at 100% identity. These 64 ASVs accounted for 43.0% of the total relative abundance in the amplicon data (Figure 6).

## 4 Discussion

Insect farming has increasingly gained attention as a source of sustainable animal protein, for waste management, valuable secondary metabolites (e.g., chitin, chitosan, and antimicrobial peptides), lipids, and insect frass as a sustainable fertiliser (Klüber et al. 2024; Caparros Megido et al. 2024; Tegtmeier, Hurka, Klüber, et al. 2021; Klüber et al. 2022; Bohm et al. 2024; Lomonaco et al. 2024; Praeg and Klammsteiner 2024; Klüber and Gabche 2025; Reyer et al. 2025). Particularly un‐ and underused side streams are a promising field of research to increase economic potential and sustainability. In this context, the microbial gut communities may play a crucial role in digestion and host health. While many studies focus on diet‐dependent variations in the gut microbiome (e.g., Tegtmeier, Hurka, Klüber, et al. 2021; Klammsteiner et al. 2021; Gorrens et al. 2022; IJdema et al. 2022; Eke et al. 2023; Silvaraju et al. 2024; Vecherskii et al. 2025; Gurusinga et al. 2026), fewer have explored the frass microbiome (Praeg and Klammsteiner 2024; Cifuentes et al. 2020; Gold et al. 2020; Fuhrmann et al. 2022). This is curious as insect frass is known to be a potent organic fertiliser and the associated microbial communities may support this. Based on amplicon sequencing and culture‐dependent data, we found feed type to strongly impact the microbial communities in BSFL frass. By comparison, thermal hygienisation had a limited effect, as hypothesised. To the best of our knowledge, this is the first study comprehensively characterising the BSFL frass microbiome under different insect feeds and upon heat treatment combining culture‐independent with culture‐dependent methods.

### 4.1 Feed Composition Effects on the Frass Microbiome

Feed type generally strongly affected microbial community composition. Further, microbial community structure partially reflects the compositional differences of the respective feeds. By characterising major components in the feed (Table 1), we defined three primary feed categories including fibre‐rich, low‐protein diets (AP), high‐protein, low‐fibre diets (CF) which optionally included a high fat content (RC), and low‐fibre, low‐protein diets (PE and PP). Frass from low‐fibre diets (CF, PE, PP, and RC) contained a higher bacterial diversity compared to frass from the high‐fibre diet (AP), which showed a higher fungal diversity and the highest fungal CFU counts (Table 4). Higher fungal diversity for corresponding BSFL gut samples from high‐fibre AP over low‐fibre CF, RC and PP diets was previously reported (Gurusinga et al. 2026). The high fungal CFUs and diversity might be caused by associations with fibrous organic material while bacteria favour more accessible carbon sources (Fontaine et al. 2003). Similar trends have been reported previously, where apple fruit tissue‐based material, comparable to AP, showed a generally high fungal diversity compared to frass of low‐fibre diets (Fuhrmann et al. 2022; Oszust and Frąc 2020; Abdelfattah et al. 2021).

Compared to the high‐fibre diet, frass samples from the low‐fibre diets were more similar. Here, *Sphingobacteriaceae* (22.3%) were the dominant bacterial taxa and *Basidiomycota* (74.5%) in the fungal fraction (Figure 1, Figure 3, Table S1). *Sphingobacteriaceae* support plant health and their antioxidant systems are involved in improving soil quality by contaminant degradation (Figueiredo et al. 2022). *Basidiomycota* occur saprobic or symbiotic and typically contribute to the degradation of organic matter (e.g., wood or litter) or form mutualistic relationships with plants (Schmidt‐Dannert 2016; Li et al. 2019; Di et al. 2020).

Matching the low pH in high‐fibre frass (AP), we found acidophilic or acid‐tolerant bacterial families, including *Rhodanobacteraceae* and *Acidobacteriaceae*, which support fibre degradation, and *Acetobacteraceae*, which are involved in acetification (Gurusinga et al. 2026; Kostka et al. 2012; Ivanova et al. 2020; Guzman and Vilcinskas 2022). The high relative abundance of the fungal phylum *Ascomycota* (Figure 3, Table S1) in apple pomace frass aligns with previous findings (Abdelfattah et al. 2016). *Ascomycota* and *Basidiomycota* are both involved in the decomposition of plant organic matter, such as polysaccharides, with *Ascomycota* showing broader enzymatic capabilities (Manici et al. 2024). Overall, the fibre content seems to be a strong driver of BSFL frass‐associated microbial members and their composition.

In concert with the fibre content, other feed components impact the microbial composition. Another determinant of microbial community composition is the protein content. Amplicon sequencing data revealed that *Enterobacteriaceae* and *Bacillaceae* were found to be relatively abundant in CF frass, and *Flavobacteriaceae* and *Alcaligenaceae* in RC frass, both from protein‐rich diets. Accordingly, *Enterobacteriaceae* and *Bacillaceae* were isolated from CF frass and *Alcaligenaceae* were more often isolated from RC frass. *Enterobacteriaceae* include pathogens such as *Salmonella* and *E. coli* (Safitri et al. 2024) but also plant growth‐promoting bacteria like *Enterobacter*, which can be involved in phytohormone production or solubilisation of micronutrients and phytate mineralisation (Ramesh et al. 2014). Members of the *Bacillaceae* family have shown several positive effects, including antagonism against antibiotic‐resistant bacteria, suppression of plant‐associated diseases, or production of antifungal metabolites (los Milagros Orberá Ratón et al. 2012; Aloo et al. 2019). Similarly, *Alcaligenaceae* are growth‐promoting rhizobacteria, contributing to plant growth through phosphorus solubilisation and phytohormone and siderophore production (Fatima et al. 2020). By comparison, frass from the low‐protein potato side streams PE and PP showed a similar community structure to each other with several shared other taxa, including the relatively abundant families *Paracoccaceae*, *Beutenbergiaceae*, and *Caulobacteraceae*.

Overall, the here‐described frass‐associated microbes, their abundance trends, and literature‐based functional capacities indicate a functional link between microbial metabolic capacities and the feed composition.

Insect frass has been proposed as a microbial bridge between the insect gut and plant rhizosphere. In a companion study assessing BSFL gut‐associated microbes from the here described experiments, Gurusinga et al. (2026) reported diet‐dependent shifts, largely driven by feed nutrient profiles, particularly fibre content. Similar trends were observed in our frass samples; however, microbiome compositions diverged markedly between gut and frass. Across all low‐fibre diets, frass exhibited significantly higher bacterial diversity than corresponding gut samples—most notably in CF and PP (increases > 71%), followed by RC (36%) and AP (10.5%)—suggesting additional microbial inputs or post‐defecation dynamics. While the BSFL gut hosts a core microbiome including *Actinomycetaceae*, *Enterobacteriaceae*, *Enterococcaceae*, and *Morganellaceae* (Tegtmeier, Hurka, Klüber, et al. 2021; Gurusinga et al. 2026), only *Microbacteriaceae* and *Saccharomycetales* were consistently detected across all frass samples (Table S1). *Microbacteriaceae*, also found in the BSFL gut (Tegtmeier, Hurka, Klüber, et al. 2021; Tegtmeier, Hurka, Mihajlovic, et al. 2021), are known for plant‐beneficial traits such as phytohormone production and soil remediation (Corretto et al. 2020; Kooienga et al. 2020). In contrast, frass‐dominant taxa like *Sphingobacteriaceae* were nearly absent in gut samples, indicating a possible preference for aerobic conditions. Gut‐dominant facultative anaerobes, such as *Enterococcaceae*, *Beutenbergiaceae*, and *Morganellaceae* declined sharply in frass. Fungal diversity patterns were more variable, showing diet‐specific shifts, including a notable replacement of *Ascomycota* by *Basidiomycota* in CF frass. These findings indicate that the gut microbiome only partially determines frass microbial composition, which also reflects microbial transfer from the feed and environmental exposure. Gut and feed likely interact dynamically throughout the rearing process, shaping both gut and frass communities.

Importantly, none of the ASVs were assigned to the domain archaea. This is in line with previous studies that showed that methanogenic archaea were absent in BSFL guts and frass (Tegtmeier, Hurka, Klüber, et al. 2021; Gurusinga et al. 2026) or relatively low abundant (Klammsteiner, Walter, et al. 2020). However, other studies have shown that methane emission from BSF farms can vary between locations and feed sources (Sánchez et al. 2015; Ermolaev et al. 2019; Mertenat et al. 2019; Parodi et al. 2020) or even found a very high relative abundance of methanogenic archaea in BSFL guts (Yang et al. 2021). Therefore, depending on the feed source, BSFL frass might have the potential for methane emission and should be investigated when new feed sources are applied. Our data suggest that BSFL frass derived from the here tested side streams does not support methane emission, which would be climate‐friendly in the context of large‐scale insect farming and the application of BSFL frass as fertiliser. Overall, our results highlight substantial differences in frass‐associated microbial composition between feed types, particularly with respect to fibre and protein content. The differences likely reflect content‐related characteristics of the diets, suggesting that the frass microbiome can be optimised by the feed choice.

### 4.2 Comparison of Culture‐Independent and Culture‐Dependent Methods

In our study we adopted a robust and conservative approach by applying stringent thresholds for matching ASVs to Sanger sequences. This cautious strategy may have influenced the results, reinforcing the robustness of our findings and providing a more reliable assessment of the culture‐dependent methods. Using a stringent (100% identity, ungapped) matching criterion, ASVs with an exact match to isolate Sanger sequences represented 43.0% of the total amplicon relative abundance (Figure 6), providing a conservative estimate of overlap between the culture‐independent and culture‐dependent datasets. This finding aligns well with BSFL gut microbiota data from (Tegtmeier, Hurka, Mihajlovic, et al. 2021).

While main taxa such as *Enterobacteriaceae* (18.7% of the isolates from all frass types) and *Bacillaceae* (15% of the isolates from all frass types) were overrepresented in the Sanger dataset, their overlap with ASVs was limited, with *Enterobacteriaceae* accounting for 3% and *Bacillaceae* less than 1%. This variation might be influenced by the selective media used in the cultivation‐based approach or the relatively small sample size of Sanger sequences.

Notably, *Sphingobacteriaceae*, another main taxon, exhibited a high relative abundance in the amplicon dataset of CF, PE, PP, and RC frass, while being absent in AP frass. In line with this, they were also frequently isolated from the same frass types exclusively (8.5% of the isolates), consistent with the amplicon data. On the other hand, *Acetobacteraceae* and *Rhodanobacteraceae* showed a high relative abundance in the amplicon dataset of AP, while being absent in all other frass types, and in line with this, they were only isolated from AP frass (2% of the isolates, respectively). *Alcaligenaceae* was one of the dominant families in RC frass, and members of this family were most frequently isolated from RC frass (35% of the isolates) (Figures 1 and 5). This indicates that our cultivation approach recovered representatives of several dominant families in these frass types.

### 4.3 Heat Treatment Effects

Efforts to control or even adjust the microbial associations in insect frass towards beneficial functions must consider legal requirements (EUCOM 1925 2021). In the EU, this primarily includes its hygienisation for 1 h at 70°C before its intended use as fertiliser following the EU Regulation 142/2011. The regulation further limits *E. coli* and *Enterococcaceae* to 1000 CFU/g and mandates the absence of *Salmonella* in 25 g of frass (EUCOM 142 2011). However, scientific data on the impact of the hygienisation on insect frass are limited (Safitri et al. 2024). While the reduction or elimination of potentially harmful microbes is crucial, particularly the effects on beneficial frass microbiome members are not addressed. Based on our data, thermal treatments influence microbial loads but have little impact on overall bacterial and fungal composition in culture‐independent analyses (Figure 1, Figure 3, Figure S2, Figure S3). However, amplification‐based data are not able to discern between dead and viable cells (Wawrik et al. 2005; Vartoukian et al. 2010; Stefani et al. 2015). Heat treatments were previously found to reduce total CFU counts by less than 1 log in BSFL frass (van Looveren et al. 2021). In comparison, our data showed heterogeneous heat treatment effects on CFU counts, which partly increased and partly decreased after 1 h at 70°C (Table 4). Importantly, across all industrial side streams and independent from heat treatments *Salmonella* and *E. coli* were not detectable in these BSFL frass samples. Consistent with previous findings (Klammsteiner, Turan, et al. 2020), *E. coli* was present in the control group CF (Tables S1 and S2). The CFU for viable *Lactobacillales* on selective media, primarily assigned to the regulatory‐monitored family *Enterococcaceae* (Table S2), exceeded regulatory limits after hygienisation, with some CFU counts even increasing. This is likely caused by members such as *Enterococcus faecium* that are known for their high heat resistance (Liu et al. 2018; Brar and Danyluk 2019). Fungal CFU were enumerated on YGC after 72 h, which captures the fraction of fungi able to grow under these conditions; slower‐growing taxa may be underrepresented. We note that the absence of yeasts or filamentous fungi on the plates used for counting is not necessarily generalisable, as their presence on uncountable plates from our dilution series cannot be excluded.

Our culture‐dependent cluster analysis revealed that only a small number of taxa formed relatively large and broadly distributed clusters, while most isolates were scattered across many small clusters. This pattern indicates that the culture‐dependent microbiome is dominated by a few families, primarily members of *Enterobacteriaceae* (two clusters: one with *Klebsiella* and one with *Citrobacter*), *Alcaligenaceae* (one cluster, *Alcaligenes*), and *Enterococcaceae* (one cluster, *Enterococcus*). However, their occurrence across frass types was not uniform, while their distribution across heat‐treatment conditions was uniform for all large clusters, except cluster 36 (*Citrobacter*), which was absent in hygienised frass samples (Figure S5; Table S2). These clusters reflect near‐identical 16S rRNA sequences and should not be interpreted as strain‐level identity.

Consistent with our hypothesis that feed type would be the dominant driver of community composition, amplicon‐based analyses showed strong feed effects on both bacterial and fungal community structure, whereas hygienisation had no discernible effect on community composition. In contrast, culture‐based counts responded variably to hygienisation across feeds and media, indicating heterogeneous, taxon‐ and substrate‐dependent effects on viability rather than a uniform reduction. Contrary to our expectation that bacteria and fungi would differ systematically in their response to hygienisation, we found no consistent evidence that fungal viability or community structure was preferentially reduced or preserved relative to bacteria. However, Tables S3.2 and S3.5 indicate that significant differences among feed types occurred more frequently in bacterial than in fungal communities, suggesting that feed type may exert a more pronounced influence on bacterial community composition in this system.

Our findings highlight the generally ambiguous results of hygienisation, consistent with previous findings (van Looveren et al. 2021), and argue for a re‐evaluation of the current frass treatment procedures to align treatment and desired microbial occurrences.

## 5 Conclusion

BSFL frass has shown great potential as a biofertiliser; however, its properties must fulfil the specified regulations. To further promote the fertilising capacities of frass, its composition and its associated microbial composition should be considered. Our data suggest that a feed‐dependent adjustment of the frass‐associated microbial communities is possible, which could be used to support the fertilisers' functionality. Some of the here described microbes are linked to such putatively plant growth‐promoting functions, including phytohormone producers like *Enterococcus* (a member of lactic acid bacteria) (Lee et al. 2015; Khanghahi et al. 2024), *Alcaligenes* (Fatima et al. 2020), and *Lysinibacillus* (Jha and Mohamed 2023). *Brevundimonas* can also solubilise phosphate (Afzal et al. 2017). Lactic acid bacteria are able to synthesise organic acids and support general nutrient solubilisation in soil. Some *Klebsiella* (Ayodele et al. 2024) and *Paenibacillus* (e.g., *P. odorifer* ) (Berge et al. 2002) strains support nitrogen fixation and cycling by converting atmospheric nitrogen into plant‐available forms.

Future research should link frass composition to its associated microbial communities and assess their combined effects on plant fertilisation. The use of specific feed substrates, such as low‐fibre, high‐protein industrial side streams, could be explored to promote beneficial microbial associations in the frass. The resulting frass should also be analysed for protein, fat, and fibre content to assess the digestibility of these side streams.

Targeted approaches may include microbial enrichment through tailored feed formulations, the application of selected microbial strains as biostimulants, and the isolation of taxa with potential plant health‐promoting properties. These efforts should be integrated with analyses of frass macro‐ and micronutrient composition. In parallel, plant growth experiments should be conducted to evaluate the fertilisation efficacy of frass and its impact on soil and rhizosphere microbiomes.

## Author Contributions

**Daniel Kreft:** investigation, Visualization, Software, Data Curation, Formal Analysis, Writing – Original Draft, Writing – Review and Editing. **Sabine Hurka:** software, Data Curation, Validation, Visualization, Formal Analysis, Writing – Original Draft, Writing – Review and Editing. **Friscasari F. Gurusinga:** investigation, Data Curation, Writing – Review and Editing. **Till Röthig:** validation, Data Curation, Formal Analysis, Writing – Original Draft, Writing – Review and Editing. **Andreas Vilcinskas:** validation, Resources, Writing – Review and Editing, Funding Acquisition. **Dorothee Tegtmeier:** project Administration, Conceptualization, Methodology, Data Curation, Funding Acquisition, Validation, Resources, Supervision, Writing – Original Draft, Writing – Review and Editing.

## Funding

This work was supported by the German Federal Ministry of Research, Technology, and Space, FKZ 031B1274; Fraunhofer‐Gesellschaft.

## Ethics Statement

The majority of the work presented in this manuscript is original and has not been previously published. A limited data set derived from a previous publication has been included for comparison and clearly labelled. All analyses and interpretations in this manuscript are original and have not been submitted elsewhere.

All authors have read and approved the final submitted version of this manuscript and consent to its submission to Environmental Microbiology (EMI). All applicable local, national, and international regulations and conventions, as well as standard scientific ethical practices, were fully adhered to and respected during the conduct of this work. We consent to the publication of this manuscript in Environmental Microbiology (EMI), should it be accepted.

## Conflicts of Interest

The authors declare no conflicts of interest.

## Supporting information


**Supplementary Figure 1** Alpha rarefaction curves for the characterisation of the (A) 16S rRNA and (B) ITS2 sequencing data based on the number of observed amplicon sequence variants (ASV) after removing non‐target sequences. Mean values from 20 iterations per step with calculated standard deviation for samples from untreated (unt) and hygienised (hyg) frass from five insect feed types (AP = apple pomace, CF = chicken feed, PP = potato pulp, PE = potato peelings, RC = rapeseed cake) are shown. Replicates share the same colour.

**Supplementary Figure 2** Bacterial community composition in *Hermetia illucens* frass across feed types and treatments. Stacked column plot depicting relative bacterial abundances on the taxonomic level genus for the different feeds (AP = apple pomace, CF = chicken feed, PP = potato pulp, PE = potato peelings, RC = rapeseed cake) in untreated (unt) and hygienised (hyg) frass samples. The 20 most abundant genera across all samples are displayed, all remaining taxa (*n* = 161) were grouped as “Others”. The relative abundances base on unrarefied data.

**Supplementary Figure 3** Fungal community composition in *H. illucens* frass across feeds and heat‐treatments. Stacked column plot depicting relative fungal abundances on phylogenetic level genus for the different feeds (AP, CF, PP, PE, RC) in untreated (unt) and hygienised (hyg) frass samples. The 20 most abundant genera across all samples are displayed, all remaining fungal genera (*n* = 51) were grouped into “Others”. The relative abundances are based on unrarefied data.

**Supplementary Figure 4** Distribution of cultured and identified bacterial genera based on 16S rRNA Sanger sequencing data from untreated (unt) and hygienised (hyg) *H. illucens* frass from five feed types (AP, CF, PP, PE, RC).

**Supplementary Figure 5** Distribution of isolates among the five most abundant sequence clusters, stacked by frass type and separated by treatment. Bars represent the frequency of isolates per cluster, colors indicate frass types (apple pomace, chicken feed, potato peelings, potato pulp, and rapeseed cake). For each cluster, the genus of the representative (longest) sequence is shown in italics at the origin of the bar.

**Data S1:** Supporting Information.

## Data Availability

Raw sequencing reads are available under the project PRJEB87687 at the European Nucleotide Archive (ENA, https://www.ebi.ac.uk/ena/).
