# Supplementary figures and images for: Towards Functional Fertilisers: Feed Composition Shapes Microbial Community Structure and Viability in Black Soldier Fly ( Hermetia illucens ) Frass
Source: Environ Microbiol. 2026 Feb 5;28(2):e70249. doi: 10.1111/1462-2920.70249 (PMC12875740; doi:10.1111/1462-2920.70249)

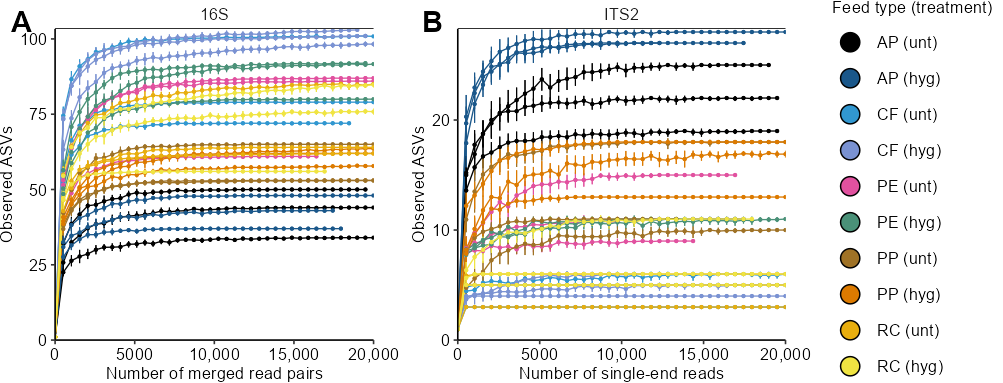

Supplement: Supplementary file 1 — Supplementary Figure 1 Alpha rarefaction curves for the characterisation of the (A) 16S rRNA and (B) ITS2 sequencing data based on the number of observed amplicon sequence variants (ASV) after removing non‐target sequences. Mean values from 20 iterations per step with calculated standard deviation for samples from untreated (unt) and hygienised (hyg) frass from five insect feed types (AP = apple pomace, CF = chicken feed, PP = potato pulp, PE = potato peelings, RC = rapeseed cake) are shown. Replicates share the same colour. [file EMI-28-e70249-s004.png]

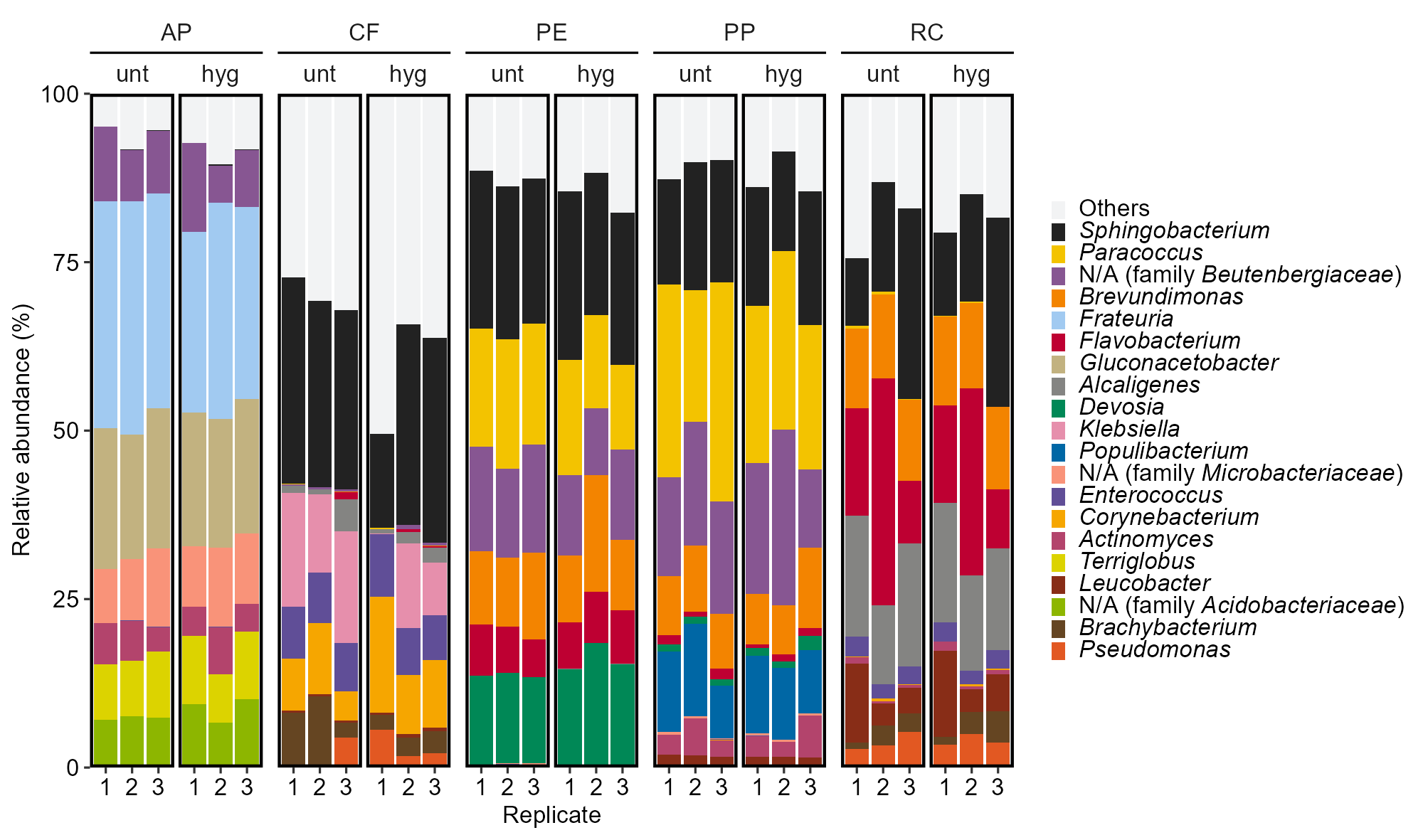

Supplement: Supplementary file 2 — Supplementary Figure 2 Bacterial community composition in Hermetia illucens frass across feed types and treatments. Stacked column plot depicting relative bacterial abundances on the taxonomic level genus for the different feeds (AP = apple pomace, CF = chicken feed, PP = potato pulp, PE = potato peelings, RC = rapeseed cake) in untreated (unt) and hygienised (hyg) frass samples. The 20 most abundant genera across all samples are displayed, all remaining taxa (n = 161) were grouped as “Others”. The relative abundances base on unrarefied data. [file EMI-28-e70249-s005.png]

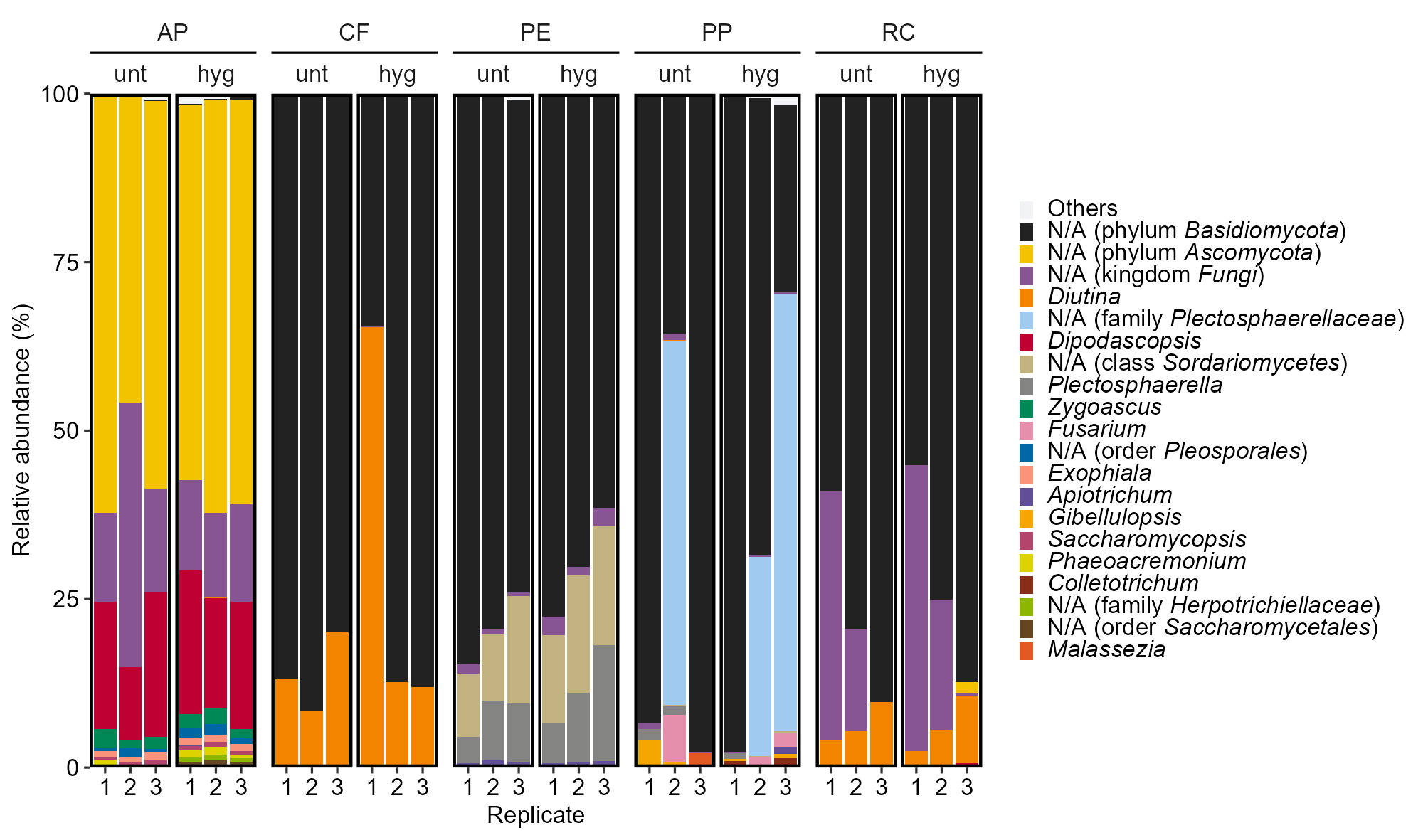

Supplement: Supplementary file 3 — Supplementary Figure 3 Fungal community composition in H. illucens frass across feeds and heat‐treatments. Stacked column plot depicting relative fungal abundances on phylogenetic level genus for the different feeds (AP, CF, PP, PE, RC) in untreated (unt) and hygienised (hyg) frass samples. The 20 most abundant genera across all samples are displayed, all remaining fungal genera (n = 51) were grouped into “Others”. The relative abundances are based on unrarefied data. [file EMI-28-e70249-s003.png]

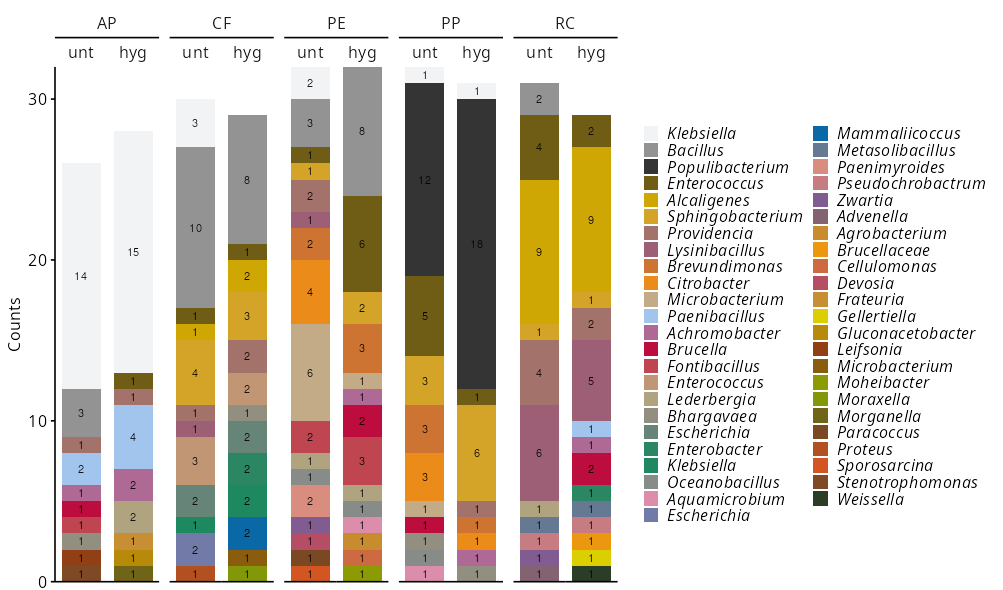

Supplement: Supplementary file 4 — Supplementary Figure 4 Distribution of cultured and identified bacterial genera based on 16S rRNA Sanger sequencing data from untreated (unt) and hygienised (hyg) H. illucens frass from five feed types (AP, CF, PP, PE, RC). [file EMI-28-e70249-s001.png]

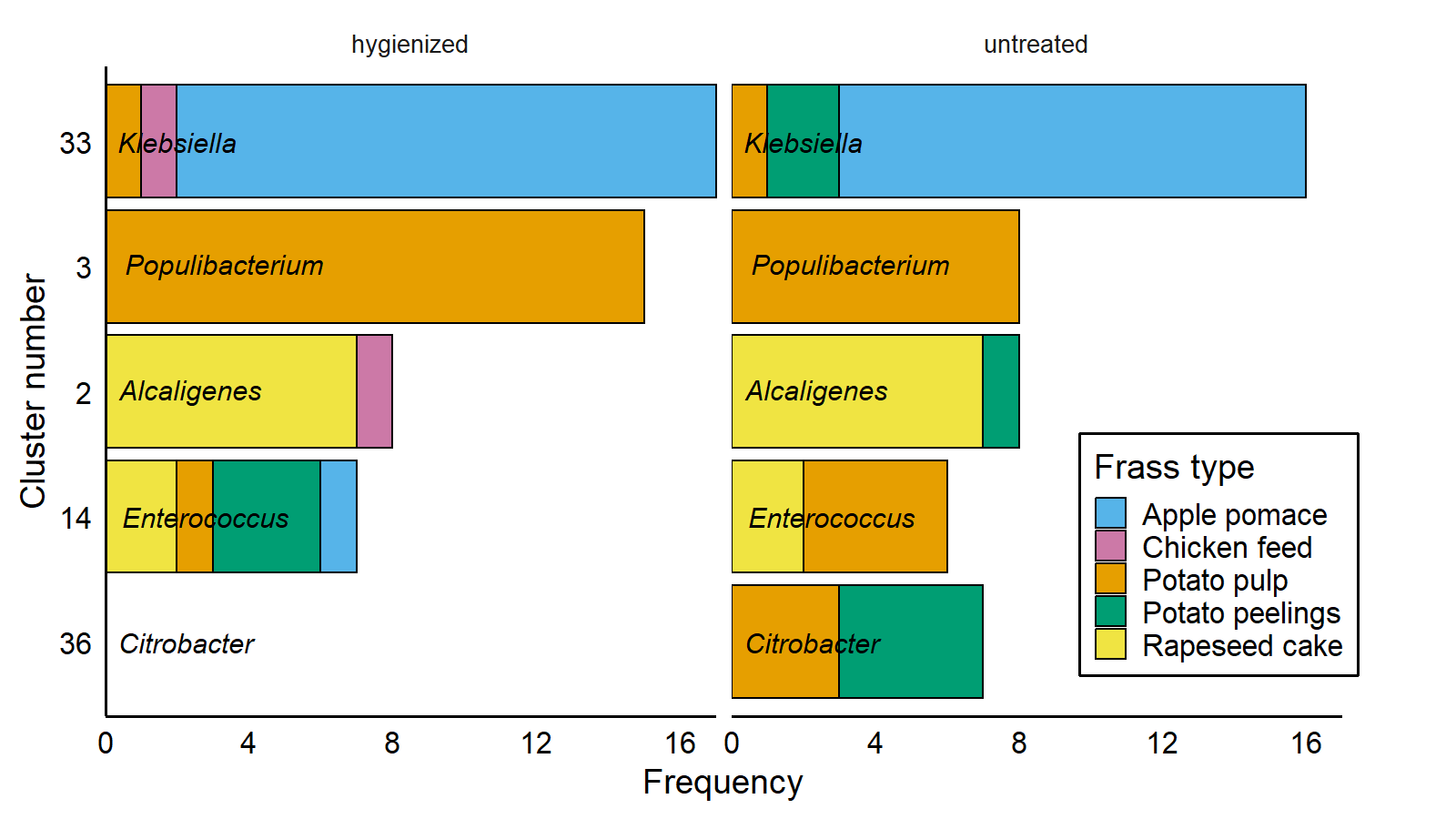

Supplement: Supplementary file 5 — Supplementary Figure 5 Distribution of isolates among the five most abundant sequence clusters, stacked by frass type and separated by treatment. Bars represent the frequency of isolates per cluster, colors indicate frass types (apple pomace, chicken feed, potato peelings, potato pulp, and rapeseed cake). For each cluster, the genus of the representative (longest) sequence is shown in italics at the origin of the bar. [file EMI-28-e70249-s006.png]
